# Comparison of the Analgesic Effects of Low-Dose Ketamine Versus Fentanyl in Patients With Long Bone Fractures in the Emergency Department: A Prospective Observational Study

**DOI:** 10.7759/cureus.46344

**Published:** 2023-10-02

**Authors:** Muhammet Yılmaz, Emre Kudu, Erkman Sanri, Sinan Karacabey, Haldun Akoglu, Arzu Denizbasi

**Affiliations:** 1 Emergency Medicine, Marmara University School of Medicine, Istanbul, TUR; 2 Emergency Medicine, Marmara University Pendik Training and Research Hospital, Istanbul, TUR

**Keywords:** trauma, low-dose ketamine, long bone fracture, fentanyl, analgesic effect

## Abstract

Aim and background

In most emergency departments (ED), opioids are the primary analgesic agents for trauma patients. However, safe alternative drugs are required because of possible adverse effects. Ketamine, an anesthetic agent, provides satisfactory analgesia at low doses and is an alternative drug that has begun to be used in numerous areas with fewer side effects. This study aimed to compare low-dose ketamine and fentanyl infusions in terms of their pain-relieving effects and observed adverse effects in patients presenting to the ED with isolated long bone fractures.

Materials and methods

This single-center observational study was conducted in the ED of the Marmara University Pendik Training and Research Hospital between August 2018 and December 2019. Patients diagnosed with isolated long bone fractures who were administered low-dose ketamine or fentanyl rapid infusions for pain relief were included in the study. Patient pain scores were evaluated using the visual analog scale (VAS) with a standard horizontal 10-centimeter line. The primary outcome of the study was to compare the changes in pain at 30 and 60 min after medication administration for each group.

Results

A total of 100 patients were included in the study. Ketamine infusion was administered to 48% (n=48) of the patients as a pain reliever. After 60 min of observation, pain was significantly reduced in both study groups. However, the pain scores at baseline (*p*=0.319), 30 min (*p*=0.631), and 60 min (*p*=0.347) after treatment were similar in both groups. In terms of the observed adverse effects, dizziness was more common in the ketamine group (p=0.010).

Conclusion

The results of this study showed that low-dose ketamine infusion (0.3 mg/kg/h) had a similar effect to fentanyl infusion (1 mcg/kg/h) as a pain reliever in patients with isolated long bone fractures in the ED.

## Introduction

Post-traumatic long bone fractures constitute 3.5-4% of emergency department (ED) admissions annually in the United States of America [1]. According to studies, traffic accidents are the leading reason with 68.1%. Falls follow this with rates between 21.8-35.1% [2]. The most frequent reason trauma patients present to the ED is severe pain [3]. If acute pain is not adequately treated, it causes many acute and chronic problems [4]. Ongoing pain and accompanying anxiety release post-injury chemicals and cause the post-injury stress response [5]. If this is not properly controlled it contributes to organ failure and trauma-related death [5]. Therefore, pain in these patients should be managed effectively.

In the article published by Hornik et al. in 2023, 2.32 million emergency visits with long bone fractures were examined [6]. While this study found no difference in analgesic use according to ethnicity or gender, it showed that 65% of the patients received analgesics and 50% of these were opioids [6]. Although opioids are effective pain relievers, they are highly addictive and have serious adverse effects such as respiratory depression, hypotension, and bradycardia [7,8]. Thus, there is a need for alternative non-opioid analgesic drugs to manage pain [9].

Ketamine is a dissociative anesthetic agent that blocks N-methyl-d-aspartate (NMDA) receptors [10]. This drug has been used as an anesthetic agent since 1970 [11]. Systematic review and meta-analysis by Karlow et al. have demonstrated that ketamine is a potent analgesic agent at subanesthetic doses [12]. When used at low doses, ketamine improves pain perception and reduces or eliminates the need for opioids [13]. In 2018, the American College of Emergency Physicians published a statement regarding low-dose ketamine [14]. Accordingly, low-dose ketamine provides effective pain relief, as well as causing less respiratory depression, and maintaining cardiac output than other analgesic agents [15]. Owing to these features, emergency physicians could use it as a safe and effective analgesic for appropriate patients.

This study aimed to compare the analgesic efficacy of the initial dose of low-dose ketamine and fentanyl (a synthetic opioid) in patients who presented to the ED with isolated post-traumatic long-bone fractures.

## Materials and methods

Study design

This single-center, prospective, observational clinical study was conducted between August 2018 and December 2019 at the ED of the Marmara University Pendik Training and Research Hospital. The hospital is an urban, medical school-affiliated, Level I trauma center and tertiary care referral center with over 200,000 adult ED presentations annually. This study was conducted in accordance with the Strengthening the Reporting of Observational Studies in Epidemiology (STROBE) guidelines [16]. Marmara University Faculty of Medicine Clinical Research Ethics Committee approved the study protocol (protocol number: 09.2018.452).

Study setting and protocol

Marmara University Pendik Training and Research Hospital is a Level I trauma center. When a trauma patient presents to our hospital, a detailed examination is performed by the primary physician, and if necessary, his/her first resuscitation and stabilization are provided. Patient pain is evaluated using the visual analog scale (VAS) score and noted in the patient file. The primary physician selects an appropriate analgesic agent in line with the current pain management guidelines, considering parameters such as pain intensity, age, and known diseases [17-19]. In patients with severe pain, potent opioids, ketamine, or a combination of these drugs are recommended for adequate analgesia [17]. If fentanyl is to be chosen to administer, it is diluted in preservative-free normal saline (NS) to a final concentration of 10 mcg/mL. Then, a rapid infusion of 1 mcg/kg is administered over 5 to 10 minutes, based on the patient's adjusted body weight [20,21]. Another agent used for adequate analgesia is ketamine, which has bolus dosing at 0.3 mg/kg (adjusted body weight) over 10 to 15 min [14]. One hour after the administration of the analgesic drug, the patient is re-evaluated for pain using the VAS score. If the patient's pain continues and the pain score cannot be reduced sufficiently (30-35% reduction in pain intensity), an additional analgesic drug is administered as a rescue agent [17,22].

Study population

Trauma patients over the age of 16 years who presented to the ED were evaluated by their primary physician, diagnosed with isolated long bone fracture, and treated with a sub-dissociative dose of ketamine (0.3 mg/kg) or fentanyl (1 mcg/kg) to relieve pain were included the study. Other inclusion criteria were having a VAS evaluation before the analgesic agent administration, acceptance of participation in the study, stable hemodynamics, no unconsciousness, and no known allergy to any of these agents. Patients who withdrew consent or required sedation during follow-up were excluded from the study.

Variables and data measurement

The patients were divided into two groups, ketamine and fentanyl, according to the analgesic agent they received. Demographic data such as age, sex, comorbidity, mechanism of injury, anatomical location of the fracture, pain intensity score, vital signs at 0, 30, and 60 min, observed adverse effects during treatment, and need for rescue analgesics were recorded for both groups.

The pain intensity of the patients was evaluated using a VAS score. A 10-cm horizontal line was used for VAS measurements. The left side of the line described “no pain,” while the right side described the “most severe pain in life.” There were no other markings on the line, and the patients marked this line based on the pain they felt. Pain was measured in centimeters from the left end of the scale to the point marked by the patient. This measurement was performed three times (before analgesic administration and 30 and 60 min after analgesic admission).

The primary outcome of our study was to compare the changes in VAS scores at the 30th and 60th minutes from baseline in both groups. Secondary outcomes included a comparison of changes in vital signs at 30 and 60 min, the need for rescue analgesics, and side effects between the two groups. According to studies in the literature, a clinically significant change is defined as a change of 1.1 to 1.37 points in the VAS score. We accepted the value of 1.37 as significant in our study [23].

Statistical analysis

The Statistical Package for Social Sciences (SPSS) for Mac 25.0 (IBM Corp., Armonk, NY) program was used for statistical analysis. The distribution normality of the data was evaluated using Kolmogorov-Smirnov test. Categorical variables are presented as numbers and percentages, whereas numerical variables are presented as medians and interquartile ranges. For comparisons between groups, we used the chi-squared test for categorical data and the Mann-Whitney U test for numerical data. Statistical significance was set at p<0.05.

## Results

**Figure 1:**
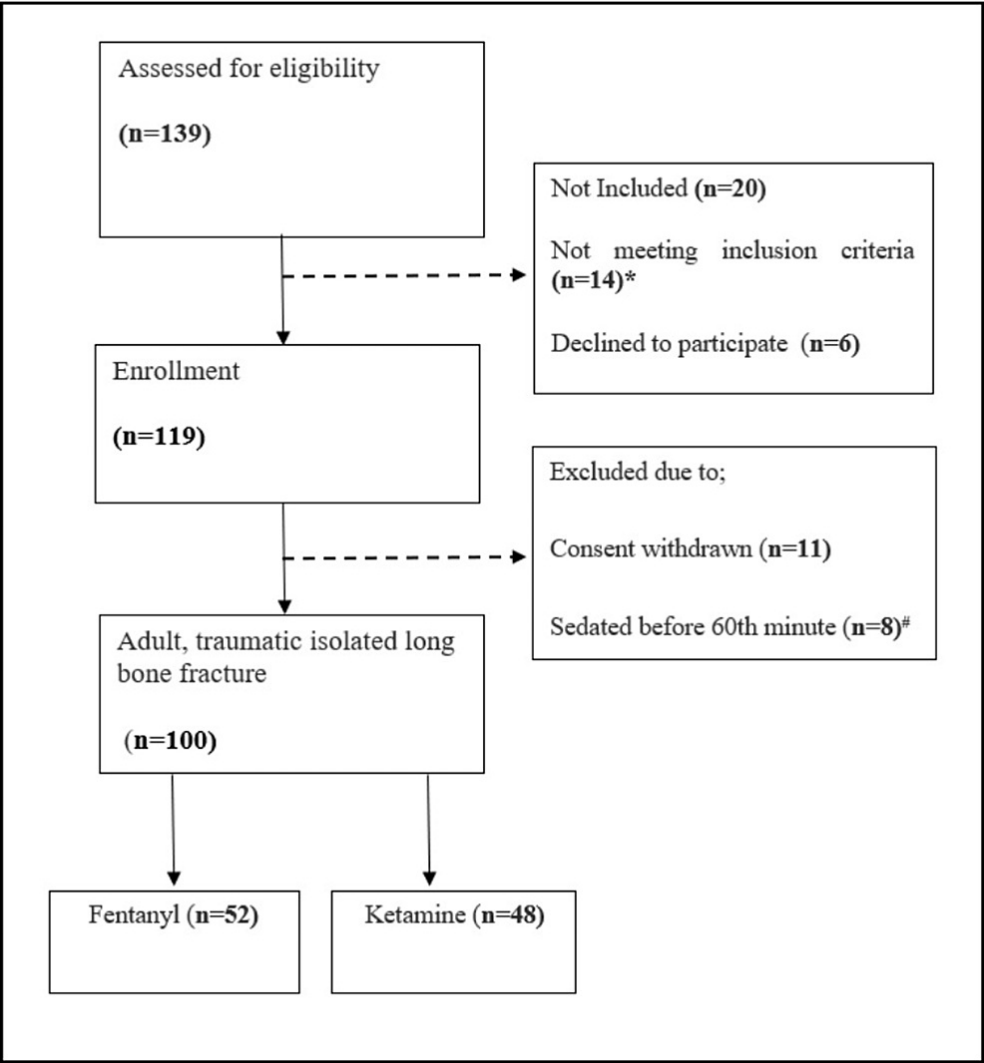
Study flow diagram * 7 patients were < 16 years old, 5 patients had unstable hemodynamics, and 2 patients were allergic to one of the agents (fentanyl or ketamine) # Sedation was applied to 8 patients for the reduction procedure

During the study period, 139 patients with isolated long bone fractures presented at the ED. Thirty-nine patients were excluded based on these exclusion criteria. One hundred patients were included in this study. It was observed that 52% of patients (n=52) received fentanyl, whereas 48% (n=48) received ketamine as an analgesic agent (Figure 1). 

The median age of the fentanyl group was 51.5 (33.0-73.7) years, and that of the ketamine group was 44.0 (30.0-60.0) years (p=0.548). Thirty percent (n=30) of the patients were female. A comparison of the demographic and fracture characteristics of the study groups is shown in Table 1.

**Table 1:** Comparison of the study groups according to demographics and fracture characteristics IQR: Interquartile range MVC: Motor vehicle collision * Pearson chi-squared ** Mann-Whitney U

	Fentanyl (n=52)	Ketamine (n=48)	Total (n=100)	p*
Age, years, median (IQR)	51.5 (33.0-73.7)	44.0 (30.0-60.0)	46.5 (32.2-65.2)	0.548**
Female sex, n (%)	15 (50.0)	15 (50.0)	30 (100.0)	0.793
Cause of injury, n (%)				
Fall	30 (57.7)	22 (42.3)	52 (100.0)	0.236
MVC	19 (54.3)	16 (45.7)	35 (100.0)	0.737
Assault	1 (25.0)	3 (75.0)	4 (100.0)	0.270
Crashing	2 (22.2)	7 (77.8)	9 (100.0)	0.610
Fracture location, n (%)				
Femur	23 (65.7)	12 (34.3)	35 (100.0)	0.044
Tibia	9 (34.6)	17 (65.4)	26 (100.0)	0.039
Fibula	40 (54.8)	33 (45.2)	73 (100.0)	0.358
Humerus	12 (44.4)	15 (55.6)	27 (100.0)	0.358
Radius	9 (52.9)	8 (47.1)	17 (100.0)	0.932
Ulna	8 (52.9)	9 (47.1)	17 (100.0)	0.654

**Figure 2:**
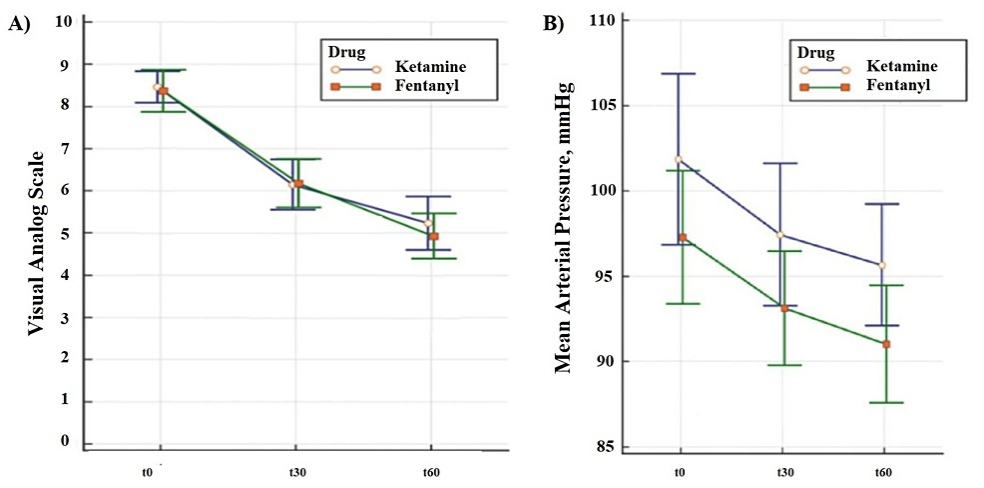
A) Alteration of VAS scores over time in both groups; B) alteration of mean arterial pressures over time in both groups Sequential measurements analysis of variance (ANOVA) A) Inter groups (ketamine vs fentanyl) p=0.682; in groups (0 vs 30 vs 60), both arms p<0.001 B) Inter groups (ketamine vs fentanyl) p=0.086; in groups (0 vs 30 vs 60), both arms p<0.001

The baseline (t0), 30th-, and 60th-minute VAS scores of both groups are shown in Table 2. In both groups, there was a decrease of approximately 3 points in the VAS scores at the end of the observation period (Figure 2-A). A similar reduction in the VAS score was observed between the groups in terms of pain relief effectiveness. The median and 95% confidence interval (CI) distributions of the baseline (t0), 30th, and 60th-minute mean arterial pressure (MAP) values of the two groups are shown in Figure 2-B.

**Table 2:** Median VAS scores in both groups IQR: Interquartile range VAS: Visual analog scale * Mann-Whitney U

Median (IQR)	Fentanyl (n=52)	Ketamine (n=48)	Total (n=100)	p*
VAS, t_0_	9.0 (7.0-10.0)	9.0 (7.3-10.0)	9.0 (7.0-10.0)	0.319
VAS, t_30_	6.0 (4.3-8.0)	6.0 (5.0-8.0)	6.0 (5.0-8.0)	0.631
VAS, t_60_	5.0 (4.0-6.0)	5.0 (4.0-7.0)	5.0 (4.0-6.8)	0.347
Δ VAS, t_0_-t_30_	2.0 (1.0-3.0)	2.0 (1.0-3.8)	2.0 (1.0-3.0)	0.759
Δ VAS, t_30_-t_60_	1.0 (0.0-2.0)	1.0 (0.0-2.0)	1.0 (0.0-2.0)	0.461
Δ VAS, t_0_-t_60_	3.5 (2.0-5.0)	3.0 (1.3-4.8)	3.0 (2.0-5.0)	0.453

The adverse effects observed throughout the study and the need for rescue agents are summarized in Table 3. The incidence of dizziness was significantly higher in the ketamine group than in the control group. No medical intervention was required for any patient with adverse effects, and the effects regressed spontaneously during follow-up. The need for rescue analgesics was lower in the fentanyl group.

**Table 3:** Adverse effects and need for rescue analgesics in the treatment groups Percentages are given as row percentages * Pearson chi-squared

	Fentanyl (n=52)	Ketamine (n=48)	Total (n=100)	p*
Hypoxia, n (%)	3 (60.0)	2 (40.0)	5 (100.0)	0.713
Dizziness, n (%)	1 (11.1)	8 (88.9)	9 (100.0)	0.010
Sleeping state, n (%)	0 (0.0)	1 (100.0)	1 (100.0)	0.296
Slowing reactions, n (%)	0 (0.0)	1 (100.0)	1 (100.0)	0.296
Nausea, n (%)	0 (0.0)	2 (100.0)	2 (100.0)	0.137
Numbness, n (%)	0 (0.0)	3 (100.0)	3 (100.0)	0.067
Rescue analgesic, n (%)	10 (33.3)	20 (66.7)	30 (100.0)	0.014

## Discussion

In the ketamine group, VAS scores decreased by 2.0 units at 30 min and 3.0 units at 60 min, whereas it decreased by 2.0 units at 30 min and 3.5 units at 60 min in the fentanyl group. The VAS scores decreased clinically significant [23] in both groups; however, there was no significant difference between them. Although dizziness was more common in the ketamine group, none of the patients required medical intervention due to adverse effects. These results demonstrate that low-dose ketamine is a promising agent for pain management in patients with long bone fractures in the ED.

Jahanian et al. investigated the efficacy and safety of low-dose ketamine and morphine [24]. In their randomized controlled trial involving 156 patients, pain scores were reduced in the ketamine group (8.28 to 4.63 at t30), similar to the morphine group (8.18 to 4.84 at t30). No significant differences were observed in terms of adverse effects [24]. In our study, ketamine was compared with fentanyl (synthetic opioid instead of morphine), and the results were similar. In addition, the need for rescue agents was evaluated by Jahanian et al. [24]. If there was no pain-relieving response to the first administered agent, the same agent was administered again at half the dose. If there was no response to the second application, fentanyl was administered. In both groups, the half-dose agent application was equal. Six patients in the ketamine group and three patients in the morphine group required fentanyl. Although there were similar decreases in the pain scores, the need for rescue agents was higher in the ketamine group [24]. Similarly, in our study, the need for a rescue agent was higher in patients in the ketamine group. Although ketamine decreases pain scores equally, additional analgesics are required in the long term.

Beaudoin et al. conducted a double-blind randomized placebo-controlled trial in patients with severe acute pain [25]. Patients were divided into three groups according to the treatment they received. While the control group received only morphine, the other two groups received additional treatment with 0.15 mg/kg and 0.30 mg/kg ketamine. The authors found that patients in the group receiving 0.3 mg/kg ketamine needed fewer rescue analgesics, but a higher proportion of dizziness was observed [25]. Similar to our study, none of these adverse effects required medical intervention.

Bronsky et al. compared ketamine and fentanyl in trauma patients with severe pain in the pre-hospital setting and found that ketamine provided adequate analgesia in more patients [26]. In addition, they did not find any significant differences in the vital parameters of patients before and after treatment. Our study found a decrease in mean arterial blood pressure. Although this change was notable, it was not statistically significant. It is believed that a decrease in pain causes this decrease physiologically [27].

Widespread use of opioids has increased the incidence of opioid-related illnesses and deaths. This problem was defined as a public health crisis in 2017 by the United States Department of Health & Human Services and continues to be updated every year [28]. Again in this direction, non-opioid pharmacological treatments have come to the fore as pain relievers [29]. In this context, the results of our study showed that ketamine is an alternative agent that reduces opioid-related problems.

This was an observational study with certain limitations. The primary physician determined the drugs administered to the patients and the infusion rate. Therefore, undesirable differences in drug distribution were observed at some fracture sites (the femur and tibia). Although no difference was detected between the initial VAS scores in both groups, the effect of this distribution on the study results remains unclear. Moreover, in this study, the infusion rates of the drugs were not evaluated. Previous studies have reported that with rapid applications of ketamine, adverse effects occur more frequently because serum concentrations reach higher levels in a short time [30]. By conducting studies examining the infusion rate, it is possible to provide accurate comments on whether the side effect of dizziness is related to the infusion rate. Finally, pain assessments were only performed at three time points (0, 30, and 60 min) for one hour. Since the main purpose of our study was to examine the effect of the initial dose of analgesics, the patients were followed up for one hour to eliminate rescue analgesic effects. However, it is also known that the effects of ketamine and fentanyl are prolonged. A longer follow-up period is recommended in future studies.

## Conclusions

Low-dose ketamine appears to be an alternative to fentanyl for pain treatment in patients with long bone fractures in the ED. Although no serious adverse effects were observed in our study, dizziness was more frequent in patients treated with ketamine. Therefore, ED physicians should be aware of these adverse effects. We believe that ketamine will be used more frequently as an alternative to opioids with future randomized controlled studies.
